# Are multifaceted interventions more effective than single-component interventions in changing health-care professionals’ behaviours? An overview of systematic reviews

**DOI:** 10.1186/s13012-014-0152-6

**Published:** 2014-10-06

**Authors:** Janet E Squires, Katrina Sullivan, Martin P Eccles, Julia Worswick, Jeremy M Grimshaw

**Affiliations:** School of Nursing, University of Ottawa, Ottawa, Canada; Clinical Epidemiology Program, Ottawa Hospital Research Institute, Centre for Practice-Changing Research (CPCR), 501 Smyth Road, Room 1282, Box 711, Ottawa, Ontario K1H 8L6 Canada; Institute of Health and Society, Newcastle University, Newcastle, UK; Cochrane Effective Practice and Organisation of Care Group, Ottawa Hospital Research Institute, Ottawa, Canada; Department of Medicine, University of Ottawa, Ottawa, Canada

## Abstract

**Background:**

One of the greatest challenges in healthcare is how to best translate research evidence into clinical practice, which includes how to change health-care professionals’ behaviours. A commonly held view is that multifaceted interventions are more effective than single-component interventions. The purpose of this study was to conduct an overview of systematic reviews to evaluate the effectiveness of multifaceted interventions in comparison to single-component interventions in changing health-care professionals’ behaviour in clinical settings.

**Methods:**

The *Rx for Change* database, which consists of quality-appraised systematic reviews of interventions to change health-care professional behaviour, was used to identify systematic reviews for the overview. Dual, independent screening and data extraction was conducted. Included reviews used three different approaches (of varying methodological robustness) to evaluate the effectiveness of multifaceted interventions: (1) effect size/dose-response statistical analyses, (2) direct (non-statistical) comparisons of multifaceted to single interventions and (3) indirect comparisons of multifaceted to single interventions.

**Results:**

Twenty-five reviews were included in the overview. Three reviews provided effect size/dose-response statistical analyses of the effectiveness of multifaceted interventions; no statistical evidence of a relationship between the number of intervention components and the effect size was found. Eight reviews reported direct (non-statistical) comparisons of multifaceted to single-component interventions; four of these reviews found multifaceted interventions to be generally effective compared to single interventions, while the remaining four reviews found that multifaceted interventions had either mixed effects or were generally ineffective compared to single interventions. Twenty-three reviews indirectly compared the effectiveness of multifaceted to single interventions; nine of which also reported either a statistical (dose-response) analysis (*N* = 2) or a non-statistical direct comparison (*N* = 7). The majority (*N* = 15) of reviews reporting indirect comparisons of multifaceted to single interventions showed similar effectiveness for multifaceted and single interventions when compared to controls. Of the remaining eight reviews, six found single interventions to be generally effective while multifaceted had mixed effectiveness.

**Conclusion:**

This overview of systematic reviews offers no compelling evidence that multifaceted interventions are more effective than single-component interventions.

**Electronic supplementary material:**

The online version of this article (doi:10.1186/s13012-014-0152-6) contains supplementary material, which is available to authorized users.

## Background

One of the greatest challenges for health-care systems globally is how to best translate research evidence into clinical practice, which includes how to change health-care professionals’ behaviours to reflect the best evidence. A commonly held view is that multifaceted interventions (i.e. an intervention with two or more components) are more effective than single-component interventions [1]. On the surface, the rationale for this widely held belief is compelling; it is well documented that there are multiple barriers at different levels to changing health-care professionals’ behaviours [2,3]. In theory, multifaceted interventions that target several of these barriers simultaneously should be more effective than single-component interventions that address just one of the many barriers to a behaviour. Yet, despite this face validity, evidence as to whether multifaceted interventions are truly more effective remains uncertain. Multifaceted interventions, by their nature, require more resources (costs) and are inherently more complex to deliver and sustain [4]. It is therefore critical to determine whether the additional resources and effort required for multifaceted interventions lead to better behavioural outcomes for health-care professionals.

Existing evidence on the effectiveness of multifaceted interventions is limited and conflicting. Early systematic reviews by Davis et al. [5] (on the effectiveness of continuing medical education) and Wensing and Grol [6] (on the effectiveness of multifaceted and single interventions in primary care) argue that multifaceted interventions are more effective than single-component interventions. However, the methods used in these studies are unclear, and there are common methodological issues in the primary studies included in the reviews such as unit of analysis errors. Additionally, synthesis in the reviews was through vote counting which comprises a weak form of indirect evidence for the effectiveness of multifaceted interventions [5,6]. More recent systematic reviews [7,8] that used robust statistical tests to investigate this topic are in opposition to these early findings. Grimshaw et al. [7] was the first review team to use robust statistical methods to explore the effectiveness of multifaceted interventions in changing health-care professionals’ behaviours; they concluded that multifaceted are not necessarily more effective than single-component interventions.

In summary, evidence of the effectiveness of multifaceted interventions in changing health-care professionals’ behaviours to reflect best practice is uncertain. The purpose of this study was to conduct an overview of systematic reviews to evaluate the effectiveness of multifaceted interventions in comparison to single-component interventions in changing health-care professionals’ behaviour in clinical settings.

## Methods

### Design

The design of this study was an overview of systematic reviews. Overviews have become increasingly popular in recent years [9]. This may be because they have potential advantages over systematic reviews. For example, one limitation of systematic reviews that can be overcome by doing an overview is that the overviews allow one to compare data on different interventions or conditions, providing a broader summary of the current information available [10,11]. Second, overviews can compare the findings of several reviews and determine reasons for conflicting reviews, allowing users to base their decisions on the most current, reliable and suitable data for their context [10,12]. For these reasons, and because we were interested in broadly evaluating the effectiveness of multifaceted interventions in comparison to single-component interventions, an overview of systematic reviews was the preferred design for this study.

### Data source

The data source for this overview was the *Rx for Change* database (www.rxforchange.ca). This database contains quality-appraised and summarized systematic reviews on the effectiveness of (1) interventions for improving prescribing by health-care professionals and medicines use by consumers and (2) professional interventions that impact the delivery of care. The *Rx for Change* database is populated using systematic methods. It is regularly updated using sensitive searches of MEDLINE, EMBASE, DARE and *The Cochrane Library* [13–15]. All reviews eligible for inclusion in the database are screened and assessed for methodological quality by two individuals on the Rx for Change team (a quality assessment is performed by one reviewer, with a second reviewer verifying the assessment). Methodological quality is assessed using AMSTAR, an 11-item valid and reliable measurement tool to assess methodological quality of systematic reviews [16].

### Inclusion criteria

Included reviews in this overview were required to explicitly report a comparison of the effectiveness of multifaceted to single-component interventions to change the behaviour of health-care professionals. A health-care professional was defined as a person who by education, training, certification or licensure is qualified to and is engaged in providing health care. Multifaceted interventions were defined using the Cochrane Effective Practice and Organisation of Care Group definition of ‘any intervention including two or more components’ [17]. Behaviour change refers to a change that reflects research evidence. Examples of such behaviour changes could be prescribing behaviours (e.g. reducing the number of prescriptions written for antibiotics), use of guidelines and improving hand hygiene. The actual behaviours will vary across individual systematic reviews. Included reviews were restricted to those rated as moderate or high methodological quality (i.e. AMSTAR rating of 4 or higher and thus summarized in the *Rx for Change* database). This decision was based on our and others experiences that it is difficult to draw meaningful conclusions based on data from low-quality reviews [13,14]. A minimum of three primary studies per review comparing multifaceted to single interventions (for direct comparisons) or comparing multifaceted interventions to a control and single interventions to a control (for indirect comparisons) was also required; this is consistent with a recent review [18] that examined the extent to which social cognitive theories explain health-care professionals’ intention to adopt a clinical behaviour. If a review was updated, only the latest version of the review was included. Systematic reviews that were published in more than one source were treated as linked reviews and only the most comprehensive paper was included. No reviews were excluded based on the type of health-care professional, the targeted behaviour (the outcome), study designs of the primary studies or publication date.

### Selection of studies and data extraction

Dual, independent screening and data extraction was conducted. Screening involved assessing the full-text articles of all moderate- and high-quality reviews that targeted health-care professionals in the *Rx for Change* database published on or before May 1, 2013. This included all reviews summarized in Rx for Change up to and including the April 2013 update (which included reviews published before April 2012) and reviews identified in the *Rx for Change* database as published between April 2013 and May 2013 but not yet summarized in the database. For included reviews, data was extracted on the following characteristics: year of publication, focus of the review, setting, population, number of primary studies, primary study designs, interventions, comparisons, outcomes and all findings related to the effectiveness of multifaceted compared to single-component interventions. Disagreements in both screening and data extraction were resolved by consensus and consultation with a third overview author when necessary.

### Data synthesis

Included reviews used three different approaches (of varying methodological robustness) to evaluate the effectiveness of multifaceted interventions. Some reviews reported more than one analytic approach; where multiple approaches were reported, all approaches were extracted and a sensitivity analysis conducted to see if overall conclusions differed when these reviews were limited to just their most robust analysis. The three analytic approaches reported, starting with the most robust, are as follows: (1) effect size/dose-response statistical analyses, (2) direct comparisons (non-statistical) of the effectiveness of multifaceted compared to single interventions and (3) indirect comparisons of the effectiveness of multifaceted compared to single interventions (by comparing multifaceted interventions to controls vs. single interventions to controls). A dose-response analysis examines whether there is a relationship between the effectiveness (the response) and the number of intervention components (the dose); effectiveness is reported statistically, frequently using the Kruskal-Wallis statistical test which assesses for differences between groups (e.g. between effectiveness of interventions with one component, two components, three components, etc.). Effectiveness in the reviews that reported non-statistical direct and indirect comparisons of multifaceted to single-component interventions was determined by vote counting. In line with a recent previous overview [14], and to increase the robustness of this analysis, reviews were categorized before vote counting as follows: (1) generally effective (if more than two thirds of its primary studies demonstrated positive effects), (2) mixed effects (if one third to two thirds of its primary studies demonstrated positive effects) and (3) generally ineffective (if fewer than one third of its primary studies demonstrated positive effects). This step was not taken in the previous overviews [5,6] on the effectiveness of multifaceted interventions that relied on vote counting. Further discussion on the strengths and limitations of this phase of our analysis can be found in the discussion of this manuscript.

### Sensitivity analyses

Two sensitivity analyses were conducted. First, for reviews that reported greater than one analytic approach to examine the effectiveness of multifaceted to single-component interventions, we assessed whether including both analyses changed our overall conclusions. To carry out this analysis, we removed the review from the least robust approach reported (e.g. removed from indirect comparisons if a direct comparison was also reported) and compared these findings to those with all reviews included. The second sensitivity analysis we conducted was to assess the impact of overlapping reviews. According to Pieper and colleagues [10], all overviews of reviews should be assessed for overlaps of primary studies, and this overlap should be reported even when it is small and unlikely to impact the conclusion of the overview. To assess overlap, we used the Wilson and Limpsey [19] approach which is comprised of two steps. First, we identified ‘significant’ overlap (defined as 25% or more primary studies in common between two reviews [19]) for all possible pairs of reviews for each analytic approach (i.e. for each of the effect size/dose-response statistical analyses, direct (non-statistical) comparisons and indirect comparisons). Second, where significant overlap was found, we removed the smaller review and compared conclusions for the analytic approach with and without the overlapping review [19]. According to Wilson and Limpsey [19], this should result in minimal overlap (less than 10% overall).

## Results

### Description of reviews

**Figure 1:**
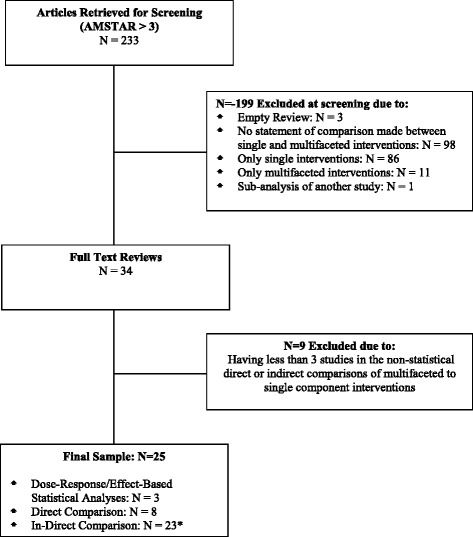
**Article screening and selection.** *Some reviews include more than one level of evidence. Therefore, the cumulative number of reviews is greater than the included number of reviews. *N* =7 of the reviews reporting indirect comparisons also reported direct comparisons, and *N* =2 of the reviews reporting indirect comparisons also reported dose-response/effect-based statistical analyses.

**Figure 2:**
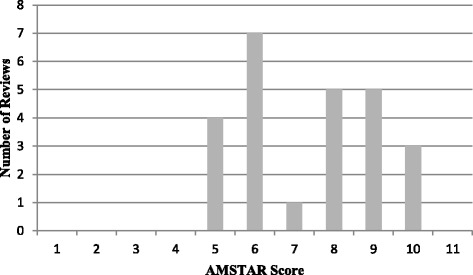
**AMSTAR scores of included reviews (** ***N*** **=25).**

Of the 233 reviews included in the *Rx for Change* database that examined professional behaviour change interventions, 25 met our inclusion criteria (Figure 1). The 25 reviews were published between 1994 and 2012. The number of primary studies per review ranged from 10 to 235, with a median of 28. Most reviews included multiple populations (physicians, nurses, pharmacists, etc.) (*N* = 24, 96%) and multiple settings (hospitals, clinics, primary care, etc.) (*N* = 23, 92%). The methodological quality of the included reviews was variable; the median AMSTAR score was 7 (range 4 to 9) (Figure 2). Several AMSTAR items were rarely reported in the included reviews: (1) providing an a priori design (working from a protocol), (2) disclosing conflict of interest for individual studies and (3) assessing publication bias.

### Sensitivity analyses

For the first sensitivity analysis, we examined whether allowing individual reviews to be considered in greater than one analytic approach changed our overall conclusions. Nine studies reported two analytic approaches; two reviews reported effect size/dose-response statistical analyses and indirect comparisons [7,8], and seven reviews [6,20–25] reported both direct and indirect comparisons. Overall, our conclusions regarding the effectiveness of multifaceted compared to single interventions did not change when these reviews were removed from the less robust (indirect comparisons) category. Therefore, the nine were retained in both analytic categories. For the second sensitivity analysis, we used the approach by Wilson and Limpsey [19] to explore the effect of overlapping reviews. However, this made no impact on our findings (see Additional file 1 for the details of this analysis). Based on this analysis, all 25 reviews were retained and summarized in this overview.

### Effectiveness of multifaceted interventions

#### Effect size/dose-response statistical analyses (*N* = 3)

Three reviews provided effect size statistical analyses of the effectiveness of multifaceted interventions [7,8,26] (Table 1). In two of these reviews, a dose-response analysis was conducted. Grimshaw et al. [7], in a review of the effectiveness of guideline dissemination and implementation interventions, constructed box plots to visually inspect the spread of effect sizes for increasing the number of intervention components. Visually, there appeared to be no relationship between the effect size and the number of components in the interventions. There was also no statistical evidence of a relationship between the number of intervention components used in the study group and the effect size (Kruskal-Wallis test, *p* =0.18 for studies with no intervention control groups and *p* =0.69 for studies with multiple intervention control groups) [7]. French et al. [8], in a review of the effectiveness of interventions to improve the appropriate use of imaging in people with musculoskeletal conditions, conducted a similar analysis. They also found that the box plots displayed no visible relationship between the effect size and the number of intervention components. Further, there was also no statistical evidence of a relationship between the number of intervention components used in the study group and the effect size (Kruskal-Wallis test, *p* value =0.48) or an increased effect size by increasing the number of intervention components (quantile regression coefficient = −2.51, 95% CI −11.58 to 6.56, *p* =0.57) [8]. Shojania et al. [26] assessed the effectiveness of computer reminders on processes and outcomes of care and compared effect sizes for single-component interventions (*N* = 18) to multifaceted interventions (*N* = 14). In their review, Shojania et al. [26] found evidence of a statistical relationship (Kruskal-Wallis test, *p* =0.04); the median improvement for single vs. usual care (with no co-interventions) was 5.7%, and for multifaceted vs. single interventions, it was only 1.9% [26].

**Table 1:** **Dose-response/effect-based statistical analysis (** ***N*** **=3)**

**First author (year) and title**	**Review characteristics**	**Review findings**	**Conclusion**
French (2010) [8]	*N*: 28 studies	Analysis based on studies with multiple intervention components as follows:	The effectiveness of multifaceted interventions did not increase incrementally with the number of components
Interventions for Improving the Appropriate Use of Imaging in People with Musculoskeletal Conditions	Study designs: randomized controlled trials, controlled trials, interrupted time series	• 1 (*N* = 11)	
		• 2 (*N* = 7)	
		• 3 (*N* = 7)	
		• 4 (*N* = 1)	
	Populations: physicians, other	There was no relationship between the effect size and the number of intervention components as evidenced by	
	Settings: primary care practices, hospitals	• No statistical evidence of a relationship between the number of interventions used in the study group and the effect size (Kruskal-Wallis test, *p* = 0.48)	
	AMSTAR (quality) score: 9	• No statistical evidence of an increased effect size by increasing the number of components (quantile regression, coefficient −2.51, 95% CI: −11.58 to +6.56, *p* = 0.57)	
Grimshaw (2004) [7]	*N*: 235 (283 papers)	Analysis based on studies with multiple intervention components as follows:	The effectiveness of multifaceted interventions did not increase incrementally with the number of components
Effectiveness and Efficiency of Guideline Dissemination and Implementation Strategies	208 studies were involved in this analysis	• 1 (*N* = 56)	
	Study designs: randomized controlled trials, controlled trials, controlled before-after, interrupted time series	• 2 (*N* = 63)	
		• 3 (*N* = 46)	
		• 4 (*N* = 28)	
		• 5 (*N* = 12)	
	Populations: physicians, nurses, pharmacists, other	• 6 (*N* = 2)	
		• 7 (*N* = 1)	
	Settings: primary care practices, hospitals, outpatient clinics, communities, nursing homes, other	There was no relationship between the effect size and the number of intervention components as evidenced by	
	AMSTAR (quality) score: 7	• For studies with no-intervention control groups, there was no statistical evidence of a relationship between the number of interventions used in the study group and the effect size (Kruskal-Wallis test, *p* = 0.18)	
		• There was no statistical evidence of a difference between studies that used multiple intervention control groups and studies with multiple intervention study groups (Kruskal-Wallis test, *p* = 0.69)	
Shojania (2009) [26]	*N*: 32 studies	Analysis based on studies with 1 intervention component (*N* = 18 studies) and 1 or more intervention components (*N* = 14 studies)	Single interventions were more effective than multifaceted interventions
The Effects of On-Screen, Point of Care Computer Reminders on Processes and Outcomes of Care	Study designs: controlled clinical trials, randomized controlled trials	There was statistical evidence of a relationship between 1 and >1 interventions used in the study group and the effect size	
	Populations: physicians	• There was a significant difference in the effect size improvement between comparisons involving single (computer reminders alone) vs. usual care (no co-interventions) and multifaceted (computer reminders plus one or more co-interventions) vs. the other interventions alone (Kruskal-Wallis test, *p* = 0.04)	
	Settings: ambulatory care settings, hospitals, nursing homes, outpatient clinics, primary care practices	• The median improvement for single vs. usual care was 5.7% (IQR: 2.0% to 24.0%)	
	AMSTAR (quality) score: 8	• The median improvement for multifaceted interventions (that is computer reminders plus additional interventions versus those additional interventions alone) was 1.9% (IQR: 0.0% to 6.2%)	

#### Direct comparisons (*N* = 8)

Eight reviews reported direct (but non-statistical) comparisons of multifaceted to single-component interventions (Table 2). Half of these reviews found multifaceted interventions to be generally effective in comparison to single-component interventions (*N* = 4/8) [20,21,23,24], while the remaining reviews found either mixed effectiveness for multifaceted interventions (*N* = 3/8) [6,25,27] or that multifaceted interventions were generally ineffective (*N* = 1/8) [22] compared to single-component interventions.

**Table 2:** **Direct comparisons (** ***N*** **=8 reviews)**

**First author (year) and title**	**Review characteristics**	**Review findings** ^**a**^	**Conclusion** ^**b**^
Beach 2006 [20]	*N*: 27 studies	3/4 studies reported multifaceted interventions to be more effective than a single intervention	Generally effective (75%)
Improving Health Care Quality for Racial/Ethnic Minorities: A Systematic Review of the Best Evidence Regarding Provider and Organization Interventions	Study designs: randomized controlled trials, clinical trials	• 1/1 study favoured multifaceted vs. reminders	
	Populations: physicians, nurses, other	• 1/1 study favoured multifaceted vs. distribution of educational materials	
	Settings: primary care practices, outpatient clinics, communities, other	• 1/2 studies favoured multifaceted vs. educational meetings	
	AMSTAR (quality) score: 5		
Hulscher (2001) [21]	*N*: 55 studies	7/8 comparisons (across *N* = 6 studies) state multifaceted interventions are more effective than single interventions	Generally effective (88%)
Interventions to Implement Prevention in Primary Care	Study designs: randomized controlled trials, controlled before-after	• 5/6 comparisons favoured multifaceted vs. group education (5 studies)	
	Populations: physicians, nurses, other	• 2/2 comparisons favoured multifaceted vs. reminders (2 studies)	
	Settings: primary care practices, outpatient clinics, medical centres		
	AMSTAR (quality) score: 5		
Jamtvedt (2006) [22]	*N*: 118 studies	6/19 studies state multifaceted interventions are more effective than single interventions (audit and feedback alone).	Generally ineffective (32%)
Audit and Feedback: Effects on Professional Practice and Health Care Outcomes	Study designs: randomized controlled trials		
	Population: any kind of health-care professional		
	Setting: any kind of organization		
	AMSTAR (quality) score: 8		
Legare (2012) [27]	*N*: 21	2/3 studies state multifaceted interventions are more effective than single interventions	Mixed effects (67%)
Patients’ Perceptions of Sharing in Decisions: A Systematic Review of Interventions to Enhance Shared Decision Making in Routine Clinical Practice	Study designs: randomized controlled trials, cluster randomized controlled trials	• 2/2 studies favoured multifaceted vs. patient mediated	
	Populations: physicians	• 0/1 study favoured multifaceted vs. educational meeting	
	Settings: primary care practices, outpatient clinics, hospitals, pharmacies, communities		
	AMSTAR (quality) score: 7		
Marinopoulos (2007) [23]	*N*: 136 studies	6/8 studies state multifaceted interventions (use of multiple media) are more effective than single interventions	Generally effective (75%)
Effectiveness of Continuing Medical Education	Study designs: randomized controlled trials, before-after, observational	• 3/5 studies favoured multifaceted over distribution of educational materials	
	Populations: physicians, pharmacists, nurses, other	• 2/2 studies favoured multifaceted over educational meetings	
	Settings: primary care practices, hospitals, long-term care facilities	• 1/1 study favoured multifaceted over audit and feedback	
	AMSTAR (quality) score: 7		
O’Brien (2007) [24]	*N*: 69 studies	12/12 studies state multifaceted interventions are more effective than single interventions	Generally effective (100%)
Educational Outreach Visits: Effects on Professional Practice and Health Care Outcomes	Study designs: randomized controlled trials	• 3/3 studies favoured multifaceted vs. audit and feedback	
	Populations: any kind of health-care professional	• 7/7 studies favoured multifaceted vs. distribution of educational materials	
	Settings: primary care practices, outpatient clinics, nursing homes, hospitals, pharmacies, communities	• 1/1 study favoured multifaceted vs. educational meetings	
		• 1/1 study favoured multifaceted vs. reminders	
	AMSTAR (quality) score: 8		
Weinmann (2007) [25]	*N*: 18 studies (in 17 papers)	2/5 studies state multifaceted interventions are more effective than single interventions (distribution of educational materials)	Mixed effects (40%)
Effects of Implementation of Psychiatric Guidelines on Provider Performance and Patient Outcome: Systematic Review	Study designs: randomized controlled trials, controlled trials, before-after		
	Populations: physicians, nurses, pharmacists, mental health clinicians, medical assistants		
	Settings: primary care practices, hospitals, communities		
	AMSTAR (quality) score: 5		
Wensing (1994) [6]	*N*: 75 studies	1/3 studies state multifaceted interventions more effective than single interventions	Mixed effects (33%)
Single and Combined Strategies for Implementing Changes in Primary Care: A Literature Review	Study designs: randomized controlled trials, controlled trials, before-after, cohort	• 0/1 study favoured multifaceted over distribution of educational materials	
	Populations: physicians	• 0/1 study favoured multifaceted over reminders	
	Settings: primary care practices	• 1/1 study favoured multifaceted over audit and feedback	
	AMSTAR (quality) score: 4		

^a^Findings are reported by the number of studies where available. In a small number of cases, reviews reported findings by the number of comparisons.

^b^Effectiveness of multifaceted compared to single-component interventions.

#### Indirect comparisons (*N* = 23)

Twenty-three reviews reported indirect comparisons of multifaceted to single-component interventions by comparing multifaceted interventions to controls and single interventions to controls (Table 3). Nine of these reviews also reported either a statistical (dose-response) analysis of the effectiveness of multifaceted interventions (*N* = 2) [7,8] or a non-statistical direct comparison of multifaceted to single-component interventions (*N* = 7) [6,20–25]. A majority (*N* = 15/23) of the reviews that reported an indirect comparison reported effectiveness data that could be categorized at the same level (i.e. as generally effective, mixed effects or generally ineffective) for both single component vs. control and for multifaceted vs. control comparisons:

- 9/23 reviews reported findings consistent with both single-component and multifaceted interventions being generally effective compared to controls [7,8,18,20,22,24,28–30]
- 5/23 reviews reported findings consistent with both single-component and multifaceted interventions having mixed effectiveness in comparison to controls [6,23,31–33]
- 1/23 reviews reported findings consistent with both single-component and multifaceted interventions being generally ineffective compared to controls [25].

**Table 3:** **Indirect comparisons of multifaceted to single interventions (** ***N*** **=23 reviews)**

**Author**	**Study characteristics**	**Review findings** ^**a**^		**Conclusion**
		**Comparison**	**Findings**	
Arnold (2005) [31]	*N*: 40 studies	Single vs. control	14/32 studies reported a single intervention was effective over a control intervention	Both multifaceted and single-component interventions have *mixed effects* when compared to controls
Interventions to Improve Antibiotic Prescribing Practices in Ambulatory Care	Study designs: randomized controlled trials, controlled before-after, interrupted time series		• 2/4 studies favoured audit and feedback vs. control	
			• 2/10 studies favoured educational meetings vs. control	
	Populations: physicians, nurses, other		• 3/8 studies favoured educational outreach vs. control	
	Settings: primary care practices, outpatient clinics, communities, other		• 2/2 studies favoured formulary vs. control	
	AMSTAR (quality) score: 7		• 2/3 studies favoured reminders vs. control	
			• 3/5 studies favour patient mediated vs. control	
			Overall: mixed effects (44%)	
		Multifaceted vs. control	4/7 studies reported a multifaceted intervention was effective over a control intervention	
			Overall: mixed effects (57%)	
Beach (2006)^b^ [20]	*N*: 27 studies	Single vs. control	8/9 studies reported a single intervention was effective over a control intervention	Both multifaceted and single-component interventions are *generally effective* when compared to controls
Improving Health Care Quality for Racial/Ethnic Minorities: A Systematic Review of the Best Evidence Regarding Provider and Organization Interventions	Study designs: randomized controlled trials, clinical trials		• 6/7 studies favoured reminders vs. control	
	Populations: physicians, nurses, other		• 1/2 studies favoured educational meetings vs. control	
	Settings: primary care practices, outpatient clinics, communities, other		• 1/1 study favoured local consensus process vs. control	
	AMSTAR (quality) score: 5		Overall: generally effective (89%)	
		Multifaceted vs. control	5/7 studies reported a multifaceted intervention was effective over a control intervention	
Boonacker (2010) [34]	*N*: 10 studies	Single vs. control	17/19 comparison (across *N* = 6 studies) reported a single intervention was effective over a control intervention	Multifaceted interventions have *mixed effects* when compared to controls, while single interventions are *generally effective* when compared to controls
Interventions in Health Care Professionals to Improve Treatment in Children with Upper Respiratory Tract Infections	Study designs: randomized controlled trials, controlled trials, controlled before-after		• 11/13 comparisons favoured reminders vs. control (3 studies)	
	Populations: physicians, nurses, pharmacists, nurse practitioners		• 4/4 comparisons favoured distribution of educational materials vs. control (2 studies)	
	Settings: primary care practices, hospitals, communities		• 2/2 comparisons favoured a local consensus process vs. control (1 study)	
	AMSTAR (quality) score: 4		Overall: generally effective (89%)	
		Multifaceted vs. control	4/6 comparisons (across *N* = 4 studies) reported a multifaceted intervention was effective over a control intervention	
			Overall: mixed effects (67%)	
Davey (2005) [28]	*N*: 69 studies	Single vs. control	24/34 studies reported a single intervention was effective over a control intervention	Both multifaceted and single-component interventions are *generally effective* when compared to controls
			• 5/6 studies favoured audit and feedback vs. control	
			• 9/11 studies favoured organizational—other vs. control	
			• 0/2 studies favoured educational outreach vs. control	
			• 5/6 studies favoured formulary vs. control	
			• 1/1 favoured professional—other vs. control	
			• 1/2 studies favoured revision of roles vs. control	
			• 3/5 studies favoured reminders vs. control	
			• 0/1 study favoured distribution of educational materials vs. control	
Interventions to Improve Antibiotic Prescribing Practices for Hospital Inpatients	Study designs: controlled trials, controlled before-after, interrupted time series		Overall: generally effective (71%)	
	Populations: physician, nurses, pharmacists, other			
	Settings: hospitals			
	AMSTAR (quality) score: 7			
		Multifaceted vs. control	18/26 studies reported a multifaceted intervention was effective over a control intervention	
			Overall: generally effective (69%)	
Flodgren (2011) [35]	*N*: 18 studies (in 19 papers)	Single vs. control	29/40 comparisons (across *N* = 8 studies) reported a single intervention (local opinion leaders) was effective over a control intervention	Multifaceted interventions have *mixed effects* when compared to controls, while single interventions are *generally effective* when compared to controls
Local Opinion Leaders: Effects on Professional Practice and Health Care Outcomes	Study designs: randomized controlled trials (cluster)			
	Populations: physicians, nurses, other		Overall: generally effective (73%)	
	Settings: primary care practices, hospitals, communities, other			
	AMSTAR (quality) score: 9			
		Multifaceted vs. control	16/26 comparisons (across *N* = 6 studies) reported a multifaceted intervention was effective over a control intervention	
			Overall: mixed effects (62%)	
Forsetlund (2009) [18]	*N*: 81 studies	Single vs. control	12/16 studies reported a single intervention was effective over a control intervention	Both multifaceted and single-component interventions are *generally effective* when compared to controls
Continuing Education Meetings and Workshops: Effects on Professional Practice and Health Care Outcomes	Study designs: randomized controlled trials		• 12/15 studies favoured educational meetings vs. control	
	Populations: nurses, pharmacists, physicians, psychiatrists, other		• 0/1 study favoured changes in structure/facilities/equipment vs. control	
	Settings: communities, hospitals, outpatient clinics, pharmacists, primary care practices		Overall: generally effective (75%)	
	AMSTAR (quality) score: 8	Multifaceted vs. control	10/14 studies reported a multifaceted intervention was effective over a control intervention	
			Overall: generally effective (71%)	
French (2010)^c^ [8]	*N*: 28 studies	Single vs. control	12/14 comparisons (across *N* = 11 studies) reported a single intervention was effective over a control intervention	Both multifaceted and single-component interventions are *generally effective* when compared to controls
Interventions for Improving the Appropriate Use of Imaging in People with Musculoskeletal Conditions	Study designs: randomized controlled trials, controlled trials, interrupted time series		• 5/6 comparisons favoured distribution of educational materials vs. control (5 studies)	
			• 5/5 comparisons favoured reminders vs.	
			control (4 studies)	
	Populations: physicians, other		• 2/3 comparisons favoured audit and feedback vs. control (2 studies)	
			Overall: generally effective (86%)	
	Settings: primary care practices, hospitals	Multifaceted vs. control	14/20 comparisons (across *N* = 16 studies) reported a multifaceted intervention was effective over a control intervention	
	AMSTAR (quality) score: 9		Overall: generally effective (70%)	
Grimshaw (2004)^c^ [7]	*N*: 235 studies (in 283 papers)	Single vs. control	53/62 comparisons (across *N* = 60 studies) reported a single intervention was effective over a control intervention	Both multifaceted and single-component interventions are *generally effective* when compared to controls
			• 7/11 comparisons favoured distribution of educational materials vs. control (11 studies)	
			• 1/1 comparison favoured educational meetings vs. control (1 study)	
			• 7/7 comparisons favoured audit and feedback vs. control (6 studies)	
			• 30/33 comparisons favoured reminders vs. control (32 studies)	
			• 1/2 comparisons favoured professional—other vs. control (2 studies)	
			• 0/1 comparisons favoured revisions of roles vs. control (1 study)	
			• 1/1 comparisons favoured continuity of care vs. control (1 study)	
			Overall: generally effective (85%)	
Effectiveness and Efficiency of Guideline Dissemination and Implementation Strategies	Study designs: randomized controlled trials, controlled trials, controlled before-after, interrupted time series			
	Populations: physicians, nurses, pharmacists, other			
	Settings: primary care practices, hospitals, outpatient clinics, communities, nursing homes, other			
	AMSTAR (quality) score: 7			
		Multifaceted vs. control	74/92 comparisons (across *N* = 78 studies) reported a multifaceted intervention was effective over a control intervention	
			Overall: generally effective (80%)	
Hakkennes (2008) [36]	*N*: 14 studies (in 27 papers)	Single vs. control	6/8 reported a single intervention was effective over a control intervention	Multifaceted interventions have *mixed effects* when compared to controls, while single interventions are *generally effective* when compared to controls
Guideline Implementation in Allied Health Professions: A Systematic Review of the Literature	Study designs: randomized controlled trials, controlled trials, controlled before-after		• 3/3 studies favoured educational meetings vs. control	
	Populations: pharmacists, other		• 1/2 studies favoured distribution of educational materials vs. control	
	Settings: hospitals, pharmacies, primary care practices, outpatient clinics, communities		• 1/1 study favoured educational outreach vs. control	
	AMSTAR (quality) score: 5		• 1/1 study favoured revision of roles vs. control	
			• 0/1 study favoured reminders vs. control	
			Overall: generally effective (75%)	
		Multifaceted vs. control	3/5 studies reported a multifaceted intervention was effective over a control intervention	
			Overall: mixed effects (60%)	
Hulscher (2001)^b^ [21]	*N*: 55 studies	Single vs. control	13/18 comparisons (across *N* = 15 studies) reported a single intervention was effective over a control intervention	Multifaceted interventions have *mixed effects* when compared to controls, while single interventions are *generally effective* when compared to controls
Interventions to Implement Prevention in Primary Care	Study designs: randomized controlled trials, controlled before-after		• 6/6 comparisons favoured audit and feedback vs. control (5 studies)	
	Populations: physicians, nurses, other		• 3/5 comparisons favoured educational meetings vs. control (4 studies)	
	Settings: primary care practices, outpatient clinics, medical centres		• 1/3 comparisons favoured distribution of educational materials vs. control (3 studies)	
	AMSTAR (quality) score: 5		• 2/3 comparisons favoured educational outreach vs. control (2 studies)	
			• 1/1 comparison favoured local consensus proves vs. control (1 study)	
			Overall: generally effective (72%)	
		Multifaceted vs. control	4/6 comparisons (across *N* = 6 studies) reported a multifaceted intervention was effective over a control intervention	
			Overall: mixed effects (67%)	
Jamtvedt (2006)^b^ [22]	*N*: 118 studies	Single vs. control	28/38 studies reported a single intervention (audit and feedback) was effective over a control intervention	Both multifaceted and single-component interventions are *generally effective* when compared to controls
Audit and Feedback: Effects on Professional Practice and Health Care Outcomes	Study designs: randomized controlled trials			
	Population: any kind of health-care professional		Overall: generally effective (74%)	
	Setting: Any kind of organization	Multifaceted vs. control	61/74 studies reported a multifaceted intervention was effective over a control intervention	
	AMSTAR (quality) score: 8		Overall: generally effective (82%)	
Laliberte (2011) [37]	*N*: 13 studies (in 16 papers)	Single vs. control	13/13 (100%) comparisons (across *N* = 6 studies) reported a single intervention was effective over a control intervention	Multifaceted interventions have *mixed effects* when compared to controls, while single interventions are *generally effective* when compared to controls
Effectiveness of Interventions to Improve the Detection and Treatment of Osteoporosis in Primary Care Settings: A Systematic Review and Meta-Analysis	Study designs: RCT, CT, other (cluster RCT)		• 12/12 comparisons favoured reminders vs. control (5 studies)	
	Population: physicians, pharmacists, other (orthopaedic surgeons)		• 1/1 comparison (1 study) favoured continuity of care vs. control	
	Setting: primary care practices, pharmacies, communities		Overall: generally effective (100%)	
	AMSTAR (quality) score: 9	Multifaceted vs. control	4/7 comparisons (across *N* = 3 studies) reported a multifaceted intervention was effective over a control intervention	
			Overall: mixed effects (57%)	
Lemmens (2009) [38]	*N*: 40 studies	Single vs. control	2/7 studies reported a single intervention was effective over a control intervention	Multifaceted interventions have *mixed effects* when compared to controls, while single interventions are *generally ineffective* when compared to controls
A Systematic Review of Integrated Use of Disease-Management Interventions in Asthma and COPD	Study designs: randomized controlled trials, controlled before-after		• 0/3 studies favoured revision roles—nursing vs. control	
	Populations: nurses, physicians and pharmacists		• 2/3 studies favoured revision roles—pharmacy vs. control	
			• 0/1 study favoured continuity of care vs. control	
			Overall: generally ineffective (29%)	
	Settings: communities, hospitals, nursing homes, outpatient clinics, pharmacies, primary care practices			
	AMSTAR (quality) score: 8			
		Multifaceted vs. control	3/7 studies reported a multifaceted intervention was effective over a control intervention	
			Overall: mixed effects (43%)	
Lloyd-Evans (2011) [29]	*N*: 11 studies	Single vs. control	3/4 comparisons (across *N* = 2 studies) reported a single intervention (educational meetings) was effective over a control intervention	Both multifaceted and single-component interventions are *generally effective* when compared to controls
Initiatives to Shorten Duration of Untreated Psychosis: Systematic Review	Study designs: randomized controlled trials, controlled trials, observational		Overall: generally effective (75%)	
	Populations: physicians, youth workers, counsellors	Multifaceted vs. control	7/10 comparisons (across *N* = 8 studies) reported a multifaceted intervention was effective over a control intervention	
	Settings: primary care practices, schools		Overall: generally effective (70%)	
	AMSTAR (quality) score: 6			
Lugtenberg (2009) [32]	*N*: 20 studies (in 30 papers)	Single vs. control	2/4 studies reported a single intervention was effective over a control intervention	Both multifaceted and single-component interventions have *mixed effects* when compared to controls
Effects of Evidence-Based Clinical Practice Guidelines on Quality of Care: A Systematic Review	Study designs: randomized controlled trials, controlled before-after, interrupted time series		• 0/1 study favoured audit and feedback vs. control	
			• 1/1 study favoured distribution of educational materials vs. control	
	Populations: physicians, other			
	Settings: primary care practices, hospitals			
	AMSTAR (quality) score: 5			
			• 1/1 study favoured educational meetings vs. control	
			• 0/1 study favoured educational outreach vs. control	
			Overall: mixed effects (50%)	
		Multifaceted vs. control	10/18 comparisons(across *N* = 16 studies) reported a multifaceted intervention was effective over a control intervention	
			Overall: mixed effects (56%)	
Marinopoulos (2007)^b^ [23]	*N*: 136 studies	Single vs. control	14/22 studies reported a single intervention was effective over a control intervention	Both multifaceted and single-component interventions have *mixed effects* when compared to controls
Effectiveness of Continuing Medical Education	Study designs: randomized controlled trials, before-after, observational		• 3/6 studies favoured distribution of educational materials vs. control	
	Populations: physicians, pharmacists, nurses, other		8/13 studies favoured educational meetings vs. control	
	Settings: primary care practices, hospitals, long-term care facilities		2/2 studies favoured educational outreach vs. control	
	AMSTAR (quality) score: 7		• 1/1 study favoured audit and feedback vs. control	
			Overall: mixed effects (64%)	
		Multifaceted vs. control	24/39 studies reported a multifaceted intervention was effective over a control intervention	
			Overall: mixed effects (62%)	
Naikoba (2001) [39]	*N*: 21 studies	Single vs. control	6/9 studies reported a single intervention was effective over a control intervention	Multifaceted interventions are *generally effective* when compared to controls, while single interventions have *mixed effects* when compared to controls
The Effectiveness of Interventions Aimed at Increasing Handwashing in Healthcare Workers - A systematic Review	Study designs: randomized controlled trials, controlled trials, observational		• 2/4 studies favoured audit and feedback vs. control	
	Populations: physicians, nurses, other		• 2/2 studies favoured reminders vs. control	
			• 1/2 studies favoured educational meetings vs. control	
	Settings: hospitals, nursing homes		• 1/1 study favoured distribution of educational materials vs. control	
	AMSTAR (quality) score: 4		Overall: mixed effects (67%)	
		Multifaceted vs. control	6/7 studies reported a multifaceted intervention was effective over a control intervention	
			Overall: generally effective (86%)	
O’Brien (2007)^b^ [24]	*N*: 69 studies	Single vs. control	26/28 studies reported a single intervention (educational outreach) was effective over a control intervention	Both multifaceted and single-component interventions are *generally effective* when compared to controls
Educational Outreach Visits: Effects on Professional Practice and Health Care Outcomes	Study designs: randomized controlled trials			
	Populations: any kind of health-care professional		Overall: generally effective (93%)	
	Settings: primary care practices, outpatient clinics, nursing homes, hospitals, pharmacies, communities	Multifaceted vs. control	40/45 studies reported a multifaceted intervention was effective over a control intervention	
	AMSTAR (quality) score: 8		Overall: generally effective (89%)	
Robertson (2010) [40]	*N*: 21 studies	Single vs. control	10/11 comparisons (across *N* = 10 studies) reported a single intervention (reminders) was effective over a control intervention	Multifaceted interventions have *mixed effects* when compared to controls, while single interventions are *generally effective* when compared to controls
The Impact of Pharmacy Computerised Clinical Decision Support on Prescribing, Clinical and Patient Outcomes: A Systematic Review of the Literature	Study designs: randomized controlled trials, controlled trials, interrupted time series, controlled before-after, cohort			
	Populations: physicians, nurses, pharmacists, nurse practitioners		Overall: generally effective (91%)	
		Multifaceted vs. control	3/9 comparisons (across *N* = 8 studies) reported a multifaceted intervention was effective over a control intervention	
	Settings: primary care practices, outpatient clinics, hospitals, pharmacies, communities			
	AMSTAR (quality) score: 4		Overall: mixed effects (33%)	
Solomon (1998) [33]	*N*: 49 studies	Single vs. control	18/34 studies reported a single intervention was effective over a control intervention	Both multifaceted and single-component interventions have *mixed effects* when compared to controls
Techniques to Improve Physicians’ Use of Diagnostic Tests: A New Conceptual Framework	Study designs: randomized controlled trials, controlled trials		• 8/15 studies favoured audit and feedback vs. control	
	Populations: physicians, nurses, medical and surgical residents		• 5/7 studies favoured distribution of educational materials vs. control	
	Settings: hospitals, outpatient clinics, communities, other		• 3/5 studies favoured reminders—general vs. control	
	AMSTAR (quality) score: 5		• 0/1 study favoured reminders—CPOE vs. control	
			• 0/4 studies favoured educational meetings vs. control	
			• 2/2 studies favoured local consensus process vs. control	
			Overall: mixed effects (53%)	
		Multifaceted vs. control	10/18 studies reported a multifaceted intervention was effective over a control intervention	
			Overall: mixed effects (56%)	
Steinman (2006) [30]	*N*: 26 studies	Single vs. control	10/10 studies reported a single intervention was effective over a control intervention	Both multifaceted and single-component interventions are *generally effective* when compared to controls
Improving Antibiotic Selection: A Systematic Review and Quantitative Analysis of Quality Improvement Strategies	Study designs: randomized controlled trials, controlled before-after, interrupted time series		• 7/7 studies favoured educational outreach vs. control	
	Populations: not specified		• 1/1 study favoured educational meetings vs. control	
	Settings: primary care practices, outpatient clinics		• 1/1 study favoured audit and feedback vs. control	
	AMSTAR (quality) score: 5		• 1/1 study favoured distribution of educational materials	
			Overall: generally effective (100%)	
		Multifaceted vs. control	21/23 studies reported a multifaceted intervention was effective over a control intervention	
			Overall: generally effective (91%)	
Weinmann (2007)^b^ [25]	*N*: 18 studies (in 17 papers)	Single vs. control	1/4 studies reported a single intervention was effective over a control intervention	Both multifaceted and single-component interventions are *generally ineffective* when compared to controls
Effects of Implementation of Psychiatric Guidelines on Provider Performance and Patient Outcome: Systematic Review	Study designs: randomized controlled trials, controlled trials, before-after		• 1/3 favoured education vs. control	
	Populations: physicians, nurses, pharmacists, mental health clinicians, medical assistants		• 0/1 favoured audit and feedback vs. control	
		Multifaceted vs. control	Overall: generally ineffective (25%)	
	Settings: primary care practices, hospitals, communities		2/8 studies reported a multifaceted intervention was effective over a control intervention	
			Overall: generally ineffective (25%)	
	AMSTAR (quality) score: 5			
Wensing (1994)^b^ [6]	*N*: 75 studies	Single vs. control	18/30 studies reported a single intervention was effective over a control intervention	Both multifaceted and single-component interventions have *mixed effects* when compared to controls
Single and Combined Strategies for Implementing Changes in Primary Care: A Literature Review	Study designs: randomized controlled trials, controlled trials, before-after, cohort		• 1/4 favoured distribution of educational materials vs. control	
	Populations: physicians		• 2/3 favoured educational outreach vs. control	
	Settings: primary care practices		• 7/10 favoured audit and feedback vs. control	
	AMSTAR (quality) score: 4		• 6/8 favoured reminders vs. control	
			• 2/5 favoured educational meetings vs. control	
			Overall: mixed effects (60%)	
		Multifaceted vs. control	7/16 studies reported a multifaceted intervention was effective over a control intervention	
			Overall: mixed effects (44%)	

^a^Findings are reported by the number of studies where available. In a small number of cases, reviews reported findings by the number of comparisons.

^b^Also in Table 2.

^c^Also in Table 1.

Of the remaining eight reviews that conducted an indirect comparison of the effectiveness of multifaceted to single-component interventions, six found single interventions to be generally effective while multifaceted had mixed effectiveness [21,34–37,40]. Another review reported that single interventions were generally effective and multifaceted were of mixed effectiveness [38], while the final review found single interventions to be of mixed effectiveness but multifaceted to be generally effective [39].

## Discussion

There has been a gradual increase in the number of studies examining the effectiveness of multifaceted interventions to change health-care professionals’ behaviour in different clinical settings. The first systematic review examining this topic was published in 1994 by Wensing and Grol [6] and included three studies that compared multifaceted to single-component interventions. Since that time, several primary studies and systematic reviews using different methods and approaches to examine the effectiveness of multifaceted interventions for different health-care professionals and clinical behaviours in diverse clinical settings have been published.

This overview draws on 25 systematic reviews of moderate or strong methodological quality to examine whether multifaceted interventions are more or less effective than single-component interventions at improving health-care professionals’ behaviours. Three approaches of varying methodological robustness were used in the included reviews to evaluate the effectiveness of multifaceted interventions: (1) effect size/dose-response statistical analyses, (2) direct comparisons (non-statistical) of the effectiveness of multifaceted compared to single interventions and (3) indirect comparisons of the effectiveness of multifaceted compared to single interventions (by comparing multifaceted interventions to controls vs. single interventions to controls). The findings of this overview do not support the commonly held assumption that multifaceted interventions are more effective than single-component interventions at changing health-care professionals’ behaviours [1]. The statistical evidence from this overview, although from a small number (*N* = 3) of reviews, indicates that increasing the number of intervention components *does not* significantly improve the effect size [7,8] and that single interventions compared to usual care may have larger effects than multifaceted compared to single interventions [26]. The majority of reviews included in this overview reported direct (but non-statistical) or indirect comparisons of the effectiveness of multifaceted compared to single-component interventions. The evidence provided in these reviews, although less robust than the statistical effect-based analyses, lends further support to the conclusion that multifaceted interventions are not necessarily more effective than single interventions. The direct comparisons had mixed results with just 4/8 reviews providing evidence that multifaceted interventions may be more effective than single interventions. With respect to indirect comparisons, most reviews found similar effectiveness for multifaceted and single interventions, and when effectiveness differed, it mostly favoured single interventions (*N* = 6/8, 75%). Thus, overall, this overview offers no compelling evidence that multifaceted interventions are more effective than single-component interventions for changing health-care professionals’ behaviours.

This overview attempted to summarize the literature on the effectiveness of multifaceted interventions in comparison to single-component interventions to provide useful information to guide researchers, knowledge translation implementers and health-care professionals to more critically consider the design and implementation of interventions to change health-care professional behaviours in different clinical settings so that effectiveness and efficiency are more appropriately balanced. If one begins with a barrier and enabler assessment to changing a specific clinical behaviour, a multifaceted intervention will often be the logical next step. However, a single-component intervention or a multifaceted intervention with fewer components might be as or even more appropriate, either as ‘the single best bet’ or as ‘the most appropriate off the shelf intervention’. We are not suggesting that multifaceted interventions are not useful, but rather that a single or less complex multifaceted intervention that is tailored to overcome the barriers and enhance the enablers of the behaviour that needs to be changed may be appropriate.

### Strengths and limitations

There are several strengths to this overview. First, it employed a comprehensive search strategy, as part of a larger project (*Rx for Change*) to examine interventions to change health-care professionals’ behaviours. This facilitated the conduct of a broad overview in a shorter period of time. Second, duplicate screening, data extraction and quality assessments were conducted. Third, a validated instrument (AMSTAR) was used to assess the methodological quality of the included reviews.

Despite the use of rigorous methods, there are also some limitations to this overview. First, we limited inclusion to reviews published in the *Rx for Change* database. This database however is large, robust and populated using systematic methods and regularly updated using sensitive searches of MEDLINE, EMBASE, DARE and *The Cochrane Library* [13,14] which limits the likelihood that we missed high-quality published systematic reviews on this topic. We did not search for grey literature, and, as such, this review may not be representative of all relevant work in the field. Second, we did not retrieve data from the primary studies that comprised the included reviews; therefore, we were limited by the information reported by the review authors. However, by focusing on the results of the systematic reviews rather than each individual primary study, we were able to obtain a broad sense of the field. Third, because of the small number of reviews reporting effect size/dose-response statistical analyses of effectiveness, we also included non-statistical assessments of effectiveness to answer our research question. This necessitated a vote counting approach to data synthesis for the non-statistical analyses. There are several weaknesses associated with using vote counting. For example, this approach to synthesis fails to account for effect sizes (vote counting gives equal weight to all associations, regardless of magnitude) and precision of the estimate from the primary studies (vote counting gives equal weight to comparisons irrespective of the sample size). Despite this limitation, our findings using vote counting support the small number of more robust statistical effect-based/dose-response statistical analyses that there is no consistent or compelling evidence that multifaceted interventions are more effective than single-component interventions. Finally, and related to the field overall, is that currently there is no generally accepted method of categorizing elements of an intervention—so, it is possible that one person’s single (composite) intervention is another person’s multifaceted intervention.

### Future research

This overview indicates several areas for future research. First, there is a lack of robust systematic statistical investigation into the effectiveness of multifaceted compared to single-component interventions. Only three (12%) of the 25 reviews in this overview reported a statistical analysis of the topic. While none of these three reviews supported improved effectiveness with more intervention components, this is a small number of cases and therefore effect-based statistical analyses to assess the effectiveness of multifaceted interventions should be replicated in future systematic reviews of behaviour change interventions. A second area for future inquiry is the assessment of cost effectiveness of multifaceted compared to less multifaceted and single interventions. Multifaceted interventions, by their nature, are likely to be more costly than single-component interventions. The added expense is frequently accepted perhaps because of the commonly held belief that multifaceted interventions are more effective than single interventions or multifaceted interventions with fewer components, which is now challenged based on the findings of this overview. While there are primary studies that examine intervention cost effectiveness generally, none of the reviews included in this overview reported cost effectiveness of multifaceted compared to single interventions; future systematic reviews of behaviour change interventions should include a summary of intervention cost effectiveness. Additionally, a focused systematic review on the cost effectiveness of multifaceted interventions broadly would also be a fruitful avenue for future inquiry.

## Conclusion

This overview of systematic reviews offers no compelling evidence that multifaceted interventions are more effective than single-component interventions as commonly believed.

Importantly, we provide systematic evidence that intervention effectiveness does not increase with more intervention components. This finding has the potential to significantly change practice by leading to less complex interventions that are less expensive and simpler to implement and thus sustain.

## Additional file

Additional file 1:
**Sensitivity analysis re-overlapping primary studies in included reviews.** This file contains the details of a sensitivity analysis conducted that assessed for the impact of overlapping primary studies across the 25 included review papers. The two-staged Wilson and Limpsey approach was used to conduct this analysis.
