# Supplementary material for: Are multifaceted interventions more effective than single-component interventions in changing health-care professionals’ behaviours? An overview of systematic reviews
Source: Implement Sci. 2014 Oct 6;9:152. doi: 10.1186/s13012-014-0152-6 (PMC4194373; doi:10.1186/s13012-014-0152-6)
Supplement: Additional file 1: — Sensitivity analysis re-overlapping primary studies in included reviews. This file contains the details of a sensitivity analysis conducted that assessed for the impact of overlapping primary studies across the 25 included review papers. The two-staged Wilson and Limpsey approach was used to conduct this analysis. [file 13012_2014_152_MOESM1_ESM.docx]

**Sensitivity Analysis re Overlapping Primary Studies in Included Reviews**

Method used follows that proposed by Wilson and Limpsey [1] as follows:

1. Calculate proportion of overlap between all pairs of reviews
2. If overlap is > 25%, remove the smaller review of the pair
3. Compare conclusions with overlapping reviews included and excluded

**Summary**

1. **Statistical Effect-Based Analysis (N=3 Reviews)**

N=0 of the 3 reviews had > 25% of its primary studies in common with a second review (see Table 5 on Page 3 of this file).

Results: no statistical evidence of improved effect size with increasing number of intervention components

1. **Direct Comparisons (N=8 Reviews)**

N=1 of the 8 reviews had > 25% of its primary studies in common with a second review (see Table 1 below). For details on all reviews in this class with any overlap, see Table 6 on Page 4 of this file)

**Table 1: Direct Comparison Reviews with > 25% Overlap of Primary Studies**

| **Review Pair** | **Number of primary studies in each review** | **Number of primary studies in common** | **Proportion of Overlap in each review** | **Review Removed for Sensitivity Analysis** |
| --- | --- | --- | --- | --- |
| Hulscher (2001) Wensing (1994) | 55 75 | 18 | 32.7 24.0 | Hulscher (2001) |

**Table 2: Sensitivity Analysis of Direct Comparison Reviews with Overlapping Review Identified in Table 2 Removed**

| **Conclusion** | **N= 8 Review (with Hulscher** **left in)** | **N=7 Reviews (with Hulscher removed)** |
| --- | --- | --- |
| Multifaceted effective compared to single | 4 (50%) | 3 (43%) |
| Multifaceted ineffective compared to single | 3 (38%) | 3 (43%) |
| Multifaceted mixed effects compared to single | 1 (13%) | 1 (14%) |

1. **Indirect Comparisons (N=23 Reviews)**

N=6 of the 23 reviews had > 25% of its primary studies in common with a second review (see Table 3 below). For details on all reviews in this class with any overlap, see Table 7 on Page 7 of this file)

**Table 3: Indirect Comparison Reviews with > 25% Overlap of Primary Studies**

| **Review Pair** | **Number of primary studies in each review** | **Number of primary studies in common** | **Proportion of Overlap in each review** | **Review Removed for Sensitivity Analysis** |
| --- | --- | --- | --- | --- |
| Grimshaw (2004)  Jamtvedt (2006) | 235 (283 papers) 118 | 30 | 10.6 25.4 | Jamtvedt (2006) |
| Arnold (2005)  Steinman (2006) | 40 26 | 13 | 32.5 50.0 | Steinman (2006) |
| Grimshaw (2004) Wensing (1994) | 235 (283 papers) 75 | 23 | 8.1 30.6 | Wensing (1994) |
| Grimshaw (2004) Hulscher (2001) | 235 (283 papers) 55 | 34 | 12.0 61.8 | Hulscher (2001) |
| Beach (2006) Grimshaw (2004) | 27 235 (283 papers) | 10 | 37.0 3.5 | Beach (2006) |
| Hulscher (2001) Wensing (1994) | 55 75 | 19 | 34.5 25.3 | Hulscher (2001) |
| French (2010) Laliberte (2011) | 28 13 (16 papers) | 6 | 21.4 37.5 | Laliberte (2011) |

**Table 4: Sensitivity Analysis of indirect Comparison Reviews with Overlapping Reviews Identified in Table 3 Removed**

| **Conclusion** | **All Reviews**  **N=23** | **Overlapped Reviews Removed**  **N=17** |
| --- | --- | --- |
| Both single component and multifaceted interventions generally effective compared to controls | 9 (39%) | 6 (35%) |
| Both single component and multifaceted interventions mixed effects compared to controls | 5 (22%) | 4 (23%) |
| Both single component and multifaceted interventions generally ineffective compared to controls | 1(4%) | 1(6%) |
| Single vs control generally effective while multifaceted vs control mixed effects | 6 (26%) | 4 (24%) |
| Single vs control generally ineffective while multifaceted vs control mixed effects | 1 (4%) | 1 (6%) |
| Single vs control mixed effects while multifaceted vs control generally effective | 1 (4%) | 1 (6%) |

**Additional Tables -- Overlap Details**

**Table 5: Statistical Effect-Based Reviews with *any* Overlap of Primary Studies**

| **Review Pair** | **Number of primary studies in the review** | **No of primary studies in common** | **Proportion of overlap in each review** |
| --- | --- | --- | --- |
| Grimshaw (2004)  French (2010) | 235 (283 papers)  28 | 1 | 0.3%  3.6%% |
| Grimshaw (2004)  Shojania (2009) | 235 (283 papers)  32 | 1 | 0.3%%  3.1% |
| French (2010)  Shojania (2009) | 28  32 | 0 | 0%  0% |

**Table 6: Direct Comparisons Reviews with *any* Overlap of Primary Studies**

| **Review Pair** | **Number of primary studies in the review** | **No of primary studies in common** | **Proportion of overlap in each review** |
| --- | --- | --- | --- |
| Hulscher (2001)  Wensing (1994) | 55  75 | 18 | **32.7%**  24.0% |
| Hulscher (2001)  Jamtvedt (2006) | 55  118 | 5 | 9.0%  4.2% |
| Hulscher (2001)  Marinopoulos (2007) | 55  136 | 6 | 10.9%  4.4% |
| Jamtvedt (2006)  O’Brien (2007) | 118  69 | 11 | 9.3%  15.9% |
| Jamtvedt (2006)  Wensing (1994) | 118  75 | 8 | 6.8%  10.7% |
| Jamtvedt (2006)  Weinmann (2007) | 118  18 | 2 | 1.7%  11.1% |
| Jamtvedt (2006)  Marinopoulos (2007) | 118  136 | 6 | 0.5%  4.4% |
| Marinopoulos (2007)  Wensing (1994) | 136  75 | 5 | 3.7%  6.7% |
| Beach (2006)  Jamtvedt (2006) | 27  118 | 1 | 3.7%  0.8% |
| Beach (2006)  Hulscher (2001) | 27  55 | 1 | 3.7%  1.8% |
| Beach (2006)  Wensing (1994) | 27  75 | 1 | 3.7%  1.3% |
| Marinopoulos (2007)  O’Brien (2007) | 136  69 | 2 | 1.5%  2.9% |
| Marinopoulos (2007)  Weinmann (2007) | 136  18 | 1 | 0.7%  5.6% |

**Table 7: Indirect Comparisons Reviews with *any* Overlap of Primary Studies**

| **Review Pair** | **Number of primary studies in the review** | **No of primary studies in common** | **Proportion of overlap in each review** |
| --- | --- | --- | --- |
| Grimshaw (2004)  Jamtvedt (2006) | 235 (283 papers) 118 | 30 | 10.6% **25.4%** |
| Jamtvedt (2006) Marinopoulos (2007) | 118 136 | 11 | 9.3% 8.1% |
| Arnold (2005) Forsetlund (2009) | 40 81 | 5 | 12.5% 6.2% |
| Grimshaw (2004) Solomon (1998) | 235 (283 papers) 49 | 11 | 3.9% 22.4% |
| Grimshaw (2004) O’Brien (2007) | 235 (283 papers) 69 | 10 | 3.5% 14.5% |
| Davey (2005) Grimshaw (2004) | 69 235 (283 papers) | 7 | 10.1% 2.5% |
| Arnold (2005)  O’Brien (2007) | 40 69 | 6 | 15% 8.7% |
| O’Brien (2007) Steinman (2006) | 69 26 | 4 | 5.8% 15.4% |
| Arnold (2005)  Steinman (2006) | 40 26 | 13 | **32.5**% **50.0**% |
| Hakkennes (2008) Robertson (2010) | 14 (27 papers) 21 | 4 | 14.8% 19.0% |
| Grimshaw (2004) Wensing (1994) | 235 (283 papers) 75 | 23 | 8.1% **30.6**% |
| Grimshaw (2004) Hulscher (2001) | 235 (283 papers) 55 | 34 | 12.0% **61.8**% |
| Forsetlund (2009) Hakkennes (2008) | 81 14 (27 papers) | 1 | 1.2% 3.7% |
| Hakkennes (2008) Lugtenberg (2009) | 14 (27 papers) 20 (30 papers) | 2 | 7.4% 6.6% |
| Lugtenberg (2009) Forsetlund (2009) | 20 (30 papers) 81 | 3 | 10.0% 3.7% |
| Hulscher (2001) Jamtvedt (2006) | 55 118 | 7 | 12.7% 5.9% |
| Solomon (1998) Wensing (1994) | 49 75 | 7 | 14.2% 9.3% |
| Grimshaw (2004) Forsetlund (2009) | 235 (283 papers) 81 | 8 | 2.8% 9.9% |
| Jamtvedt (2006) O’Brien (2007) | 118 69 | 12 | 10.2% 17.4% |
| Forsetlund (2009) Marinopoulos (2007) | 81 136 | 17 | 20.9% 12.5% |
| Forsetlund (2009) Hulscher (2001) | 81 55 | 4 | 4.9% 7.3% |
| Grimshaw (2004) Marinopoulos (2007) | 235 (283 papers) 136 | 13 | 4.6% 9.5% |
| Hulscher (2001) Marinopoulos (2007) | 55 136 | 5 | 9.1% 3.7% |
| Beach (2006) Grimshaw (2004) | 27 235 (283 papers) | 10 | **37.0**% 3.5% |
| Beach (2006) Hulscher (2001) | 27 55 | 6 | 22.2% 10.9% |
| Boonacker (2010) Steinman (2006) | 10 26 | 2 | 20% 7.7% |
| Hulscher (2001) Wensing (1994) | 55 75 | 19 | **34.5**% **25.3**% |
| Arnold (2005)  Boonacker (2010) | 40 10 | 1 | 2.5% 10% |
| Hulscher (2001) O’Brien (2007) | 55 69 | 1 | 1.8% 1.4% |
| O’Brien (2007) Wensing (1994) | 69 75 | 1 | 1.4% 1.3% |
| Jamtvedt (2006) Solomon (1998) | 118 49 | 5 | 4.2% 10.2% |
| Hakkennes (2008) Jamtvedt (2006) | 14 (27 papers) 118 | 1 | 3.7% 0.8% |
| Arnold (2005)  Grimshaw (2004) | 40 235 (283 papers) | 3 | 7.5% 1.1% |
| Grimshaw (2004) Steinman (2006) | 235 (283 papers) 26 | 2 | 0.7% 7.7% |
| Davey (2005) Robertson (2010) | 69 21 | 1 | 1.4% 4.7% |
| French (2010) O’Brien (2007) | 28 69 | 1 | 3.6% 1.4% |
| Jamtvedt (2006) Wensing (1994) | 118 75 | 8 | 6.8% 10.7% |
| Forsetlund (2009) Wensing (1994) | 81 75 | 3 | 3.7% 4.0% |
| French (2010) Jamtvedt (2006) | 28 118 | 6 | 21.4% 5.1% |
| Flodgren (2011)  Grimshaw (2004) | 18 (19 papers) 235 (283 papers) | 3 | 15.8% 1.1% |
| Flodgren (2011)  Marinopoulos (2007) | 18 (19 papers) 136 | 2 | 10.5% 1.5% |
| Marinopoulos (2007) Wensing (1994) | 136 75 | 7 | 5.1% 9.3% |
| Forsetlund (2009) Jamtvedt (2006) | 81 118 | 8 | 9.9% 6.8% |
| French (2010) Laliberte (2011) | 28 13 (16 papers) | 6 | 21.4% **37.5**% |
| Forsetlund (2009) O’Brien (2007) | 81 69 | 2 | 2.5% 2.9% |
| Arnold (2005)  Jamtvedt (2006) | 40 118 | 5 | 12.5% 4.2% |
| Jamtvedt (2006) Lugtenberg (2009) | 118 20 (30 papers) | 1 | 0.8% 3.3% |
| Lugtenberg (2009) O’Brien (2007) | 20 (30 papers) 69 | 1 | 3.3% 1.4% |
| Grimshaw (2004) Weinmann (2007) | 235 (283 papers) 18 (17 papers) | 2 | 0.7% 11.8% |
| Weinmann (2007) Jamtvedt (2006) | 18 (17 papers) 118 | 2 | 11.8% 1.7% |
| Arnold (2005)  Marinopoulos (2007) | 40 136 | 2 | 5.0% 1.5% |
| Flodgren (2011)  Jamtvedt (2006) | 18 (19 papers) 118 | 4 | 21.1% 3.4% |
| Grimshaw (2004) Lugtenberg (2009) | 235 (283 papers) 20 (30 papers) | 3 | 1.1% 10% |
| Jamtvedt (2006) Steinman (2006) | 118 26 | 3 | 2.5% 11.5% |
| Boonacker (2010) Marinopoulos (2007) | 10 136 | 2 | 20.0% 1.5% |
| Steinman (2006) Wensing (1994) | 26 75 | 2 | 7.7% 2.7% |
| Flodgren (2011)  Forsetlund (2009) | 18 (19 papers) 81 | 1 | 5.3% 1.2% |
| Boonacker (2010) Forsetlund (2009) | 10 81 | 1 | 10.0% 1.2% |
| Flodgren (2011)  French (2010) | 18 (19 papers) 28 | 1 | 5.3% 3.6% |
| Beach (2006) Jamtvedt (2006) | 27 118 | 1 | 3.7% 0.8% |
| Boonacker (2010) Grimshaw (2004) | 10 235 (283 papers) | 1 | 10% 0.4% |
| Marinopoulos (2007)  O’Brien (2007) | 136 69 | 2 | 1.5% 2.9% |
| Beach (2006) Wensing (1994) | 27 75 | 1 | 3.7% 1.3% |
| French (2010) Grimshaw (2004) | 28 235 (283 papers) | 1 | 3.6% 0.4% |
| Lemmens (2009) O’Brien (2007) | 40 69 | 1 | 2.5% 1.4% |
| Grimshaw (2004) Robertson (2010) | 235 (283 papers) 21 | 1 | 0.4% 4.8% |
| Forsetlund (2009) O’Brien (2007) | 81 69 | 1 | 1.2% 1.4% |
| Arnold (2005)  Wensing (1994) | 40 75 | 1 | 2.5% 1.3% |
| Marinopoulos (2007) Solomon (1998) | 136 49 | 1 | 0.7% 2.0% |
| Marinopoulos (2007) French (2010) | 136 28 | 2 | 1.5% 7.1% |
| French (2010) Forsetlund (2009) | 28 81 | 3 | 10.7% 3.7% |
| Forsetlund (2009) Lugtenberg (2009) | 81 20 (30 papers) | 1 | 1.2% 3.3% |
| Forsetlund (2009) Weinmann (2007) | 81 18 (17 papers) | 2 | 2.5% 11.8% |
| Davey (2005) O’Brien (2007) | 69 69 | 2 | 2.9% 2.9% |
| Flodgren (2011)  Wensing (1994) | 18 (19 papers) 75 | 1 | 5.3% 1.3% |
| Hakkennes (2008) O’Brien (2007) | 14 (27 papers) 69 | 1 | 3.7% 1.4% |
| Lemmens (2009) Robertson (2010) | 40 21 | 1 | 2.5% 4.8% |
| Weinmann (2007) Marinopoulos (2007) | 18 (17 papers) 136 | 1 | 5.9% 0.7% |

**Reference**

1. Wilson DB, Lipsey MW: **The role of method in treatment effectiveness research: Evidence from meta-analysis.** *Psychol Methods* 2001, **6:** 413-429.
